# Regarding: “Efficacy and safety of methylene blue for postoperative pain of haemorrhoids: a systematic review and meta-analysis”

**DOI:** 10.1080/07853890.2025.2590834

**Published:** 2025-11-20

**Authors:** Hawkar A. Nasralla, Omar H. Ghalib, Fahmi H. Kakamad

**Affiliations:** ^a^ Scientific Affairs Department, Smart Health Tower, Sulaymaniyah, Iraq; ^b^ College of Medicine, University of Sulaimani, Sulaymaniyah, Iraq; ^c^ Kscien Organization for Scientific Research (Middle East Office), Sulaymaniyah, Iraq

To the editor

We read with great interest the study by Zhu et al. investigating the efficacy and safety of methylene blue (MB) for postoperative pain management following haemorrhoidectomy. The authors conclude that intradermal or subcutaneous injection of methylene blue significantly reduces early postoperative pain and analgesic requirements without increasing complication rates [1]. While the topic is clinically relevant, we identified two key methodological issues that merit further consideration.

First, the meta-analysis includes trials in which the primary outcome is postoperative pain, as measured by Visual Analogue Scale (VAS) scores. In the interventions where a dye such as MB is injected and visibly colours tissues, effective blinding of patients and possibly assessors becomes challenging. Empirical meta-epidemiological work shows that trials lacking adequate blinding, particularly of patients and outcome assessors, tend to exaggerate effect sizes: for instance, a recent study found that non-blinded assessors produced a pooled ratio of odds ratios (ROR) of 0.71 (95% CI: 0.55–0.92) compared to blinded assessors, suggesting a 29% exaggeration of treatment effect [2]. Another investigation found that inadequate patient blinding was associated with a mean difference in effect size of 0.12–0.19 in trials of oral health interventions [3]. In this context, the absence of an explicit discussion of how visible staining may have compromised blinding, and thus biased subjective pain reporting, represents a key limitation that deserves acknowledgment.

Second, the included RCTs vary markedly in their control arms: some compare MB to saline/placebo, whereas others compare MB to active local anaesthetics or standard analgesic regimens. In meta-analyses of postoperative pain therapies, such heterogeneity of comparator interventions has been shown to impair interpretability: for example, one survey of 61 meta‐analyses in acute postoperative pain found that many did not adequately explore or report differences in type of surgery, comparator interventions, or analgesic background, and recommended deeper investigation of clinical heterogeneity [4]. Without prespecified subgroup analyses stratified by control type, the pooled estimate may misrepresent the incremental analgesic effect of MB relative to standard care. Clarification of how comparator heterogeneity was addressed (or why it could not be) would therefore strengthen the conclusions.

## Data Availability

Data sharing is not applicable to this article as no new data were created or analyzed in this study.
